# Amino Acid Depletion Reveals Strain- and Nitrogen Source-Dependent Physiological Responses in *Brettanomyces bruxellensis*

**DOI:** 10.3390/jof12090636

**Published:** 2026-08-26

**Authors:** Camila G-Poblete, Sandra Moreira-Ramos, Diego Rojas, Nachla Rojas-Torres, Jorge Saavedra, María Angélica Ganga

**Affiliations:** 1 Departamento de Ciencia y Tecnología de los Alimentos, Facultad Tecnológica, Universidad de Santiago de Chile (USACH), Alameda 3363, Estación Central, Santiago 9160000, Chile; camila.gonzalezpo@usach.cl (C.G.-P.); sandra.moreira.r@gmail.com (S.M.-R.); di3go.ars@gmail.com (D.R.); nachla.rojas@usach.cl (N.R.-T.); 2 DataChem Analytics, Valparaiso 2340000, Chile; jorge.saavedra@datachem.cl

**Keywords:** *Brettanomyces bruxellensis*, fungal nitrogen metabolism, apparent amino acid depletion, strain-dependent physiology, non-*Saccharomyces* yeasts

## Abstract

Fungal nitrogen metabolism is a central determinant of yeast growth, fermentative performance, and secondary metabolism in fungi associated with fermented environments. For *Saccharomyces cerevisiae*, the assimilation of nitrogen sources found in grape must, including ammonium, free amino acids, and peptides, has been widely characterized, establishing a consumption hierarchy in which proline is considered a non-preferential nitrogenous source. However, the regulation and hierarchy of nitrogen-source utilization in non-conventional fungi remain poorly characterized, particularly in yeasts adapted to anthropized fermentative niches. In this context, *Brettanomyces bruxellensis* represents a useful fungal model to study the different nitrogen sources that are used, strain-dependent physiology, and survival under nutrient-limited fermentative conditions. We hypothesized that apparent depletion of amino acid and the expression of selected amino acid permease genes are strain-dependent traits linked to the survival of *B. bruxellensis*. In this study, we evaluated the strain-dependent physiology and amino acid depletion patterns of two *B. bruxellensis* strains from wine (LAMAP2480 and LAMAP1359). In both cases, the highest apparent depletion values under the 20 amino acid condition were observed for arginine, glutamine, tryptophan and proline, indicating that these compounds contributed substantially to the organic nitrogen pool under the assayed conditions. Only LAMAP2480 isolate was able to grow in the condition without added amino acids, indicating strain and nitrogen source dependence under limited organic nitrogen availability. Transcriptional analysis of *GAP1*, *GNP1*, and *TAT1* permeases showed expression patterns dependent on both the strain and the available nitrogen source. This is the first study to combine the evaluation of physiological performance, amino acid consumption, apparent nitrogen depletion derived from amino acids normalized to OD_600_ (OD-AA-N depletion), and the transcriptional responses of selected amino acid permease genes. This knowledge may contribute to understanding the survival of *B. bruxellensis* in fermentation environments with limited nutritional resources.

## 1. Introduction

Nitrogen is a critical nutrient that directly affects yeast growth, fermentation kinetics, and the organoleptic properties of fermented beverages [1,2]. In grape must, amino acids may account for 60–90% of the nitrogen fraction, with arginine, glutamine, proline, alanine, glutamic acid, threonine, and serine commonly being the most abundant [3,4,5]. Yeasts can utilize almost thirty different compounds that contain nitrogen. These compounds can have different effects on cellular development, with there being a direct link between nitrogen availability in the culture medium and biomass production [6]. Consumption order of nitrogen sources is related to the gene expression involved in the incorporation and assimilation processes, which are generally controlled by regulatory internal mechanisms that finally determine the order in question. This allows cells to control the appropriate usage of nitrogen sources, with this being the basis of the nitrogen catabolite repression mechanism (NCR) [7]. Under preferred nitrogen sources, NCR represses the genes encoding permeases and the catabolic enzymes required for the uptake and utilization of secondary nitrogen sources [8]. Conversely, when preferred nitrogen sources are absent or depleted, repression is relieved and genes encoding the transporters and enzymes involved in the uptake and catabolism of secondary nitrogen sources can be expressed [9]. For *S. cerevisiae*, the main fermentative yeast, the major regulation mechanisms have been studied; this contrasts with what was observed in non-*Saccharomyces* yeasts, whose characterization is associated with the nitrogen utilization profile [10]. *Brettanomyces bruxellensis* is one of the main spoilage non-*Saccharomyces* yeasts in the wine industry because its presence has a negative organoleptic effect on the product [11]. Large-scale population studies have shown that this species is structured into genetically differentiated subpopulations associated with substrate origin, geographic distribution, and ploidy level, including diploid, autotriploid, and allotriploid lineages [12,13,14].

Despite the ecological and technological relevance of *B. bruxellensis*, the hierarchy of nitrogen source utilization and the transcriptional responses associated with amino acid transport remain poorly understood in this non-conventional yeast. Available evidence is restricted to a small number of studies, most of which inferred amino acid utilization from growth rather than directly quantifying extracellular depletion. Conterno et al. [15] reported growth on arginine and proline, whereas Blomqvist et al. [16] identified arginine and lysine as the nitrogen sources supporting the greatest number of cell divisions under anaerobic conditions. Crauwels et al. [17] subsequently demonstrated strain-dependent variation in amino acid utilization. Parente et al. [18] identified glutamine as a preferred nitrogen source and showed that the expression of the putative *GAP1*, *GDH1*, *GDH2*, and *GLT1* genes involved in amino acid transport and central nitrogen metabolism varied according to nitrogen source and oxygen availability. Cajueiro et al. [19] further proposed that glutamine functions as an intracellular signal regulating NCR. Nevertheless, these findings derive from a limited number of strains and experimental conditions and have not yet established a general hierarchy of amino acid consumption in *B. bruxellensis*. This knowledge gap is particularly relevant because *B. bruxellensis* survives in anthropized fermentative environments, where nitrogen limitation, ethanol accumulation, low pH, and microbial competition may act as selective pressures. Therefore, this species provides a useful fungal model to investigate how nitrogen source contributes to strain-dependent adaptation in nutrient-limited niches.

This study aimed to identify the strain-specific nitrogen utilization phenotypes of *B. bruxellensis* by integrating growth kinetics, quantitative profiles of apparent extracellular amino acid depletion, and the expression of the putative amino acid permease genes *GAP1*, *GNP1*, and *TAT1* under defined organic nitrogen conditions. This research is one of the first integrated analyses to combine growth kinetics, transcriptional responses, apparent amino acid depletion profile, and apparent amino acid-derived nitrogen depletion normalized to OD_600_ (OD-AA–N depletion) in the early- and mid-lag phases, revealing differences between the two *B. bruxellensis* strains. This has led to the understanding that apparent amino acid depletion is strain-dependent and does not appear to be a species-specific metabolic behavior.

## 2. Materials and Methods

### 2.1. Strain and Maintenance

Of the *B. bruxellensis* strains, LAMAP2480 was obtained from Chilean wine (Alto Jahuel (33°44′01″ S; 70°41′03″ O) and LAMAP1359 from Argentine wine (Mendoza 32°53′23″ S; 68°50′40″ O). Both strains belong to the culture collection of the Laboratory of Applied Microbiology and Biotechnology, Universidad de Santiago de Chile (LAMAP-USACH), and they have shown phenotypic differences [20,21]. For this study, the strains were maintained on YPD agar supplemented with CaCO_3_ (10 g/L yeast extract, 20 g/L glucose, 20 g/L peptone, 5 g/L CaCO_3_, and 20 g/L agar–agar) [22]. When necessary, each strain was precultured in 5 mL of YPD medium for 5 days at 28 °C with orbital shaking.

### 2.2. Evaluation of the Growth Kinetics of B. bruxellensis Strains

A density of 1 × 10^7^ cells/mL (20 μL) was inoculated in 2 mL of yeast nitrogen base medium without amino acids (YNB), supplemented with 20 g/L of glucose (ammonium sulfate (5 g/L) as the inorganic nitrogen source, along with biotin (2 μg/L), calcium pantothenate (40 μg/L), folic acid (2 μg/L), inositol (2 mg/L), nicotinic acid (400 μg/L), *p*-aminobenzoic acid (200 μg/L), pyridoxine HCl (400 μg/L), riboflavine (200 μg/L), thiamine HCl (400 μg/L), boric acid (500 μg/L), copper sulfate (40 μg/L), potassium iodide (100 μg/L), ferric chloride (200 μg/L), manganese sulfate (400 μg/L), sodium molybdate (200 μg/L), zinc sulfate (400 μg/L), monobasic potassium phosphate (1 g/L), magnesium sulfate (0.5 g/L), sodium chloride (0.1 g/L), calcium chloride (0.1 g/L)) (Difco, Detroit, MI, USA).

This medium was supplemented with 20 amino acids (grape must): L-glutamic acid (120.40 mg/L), L-aspartic acid (44.50 mg/L), L-histidine (50.00 mg/L), L-lysine (24.00 mg/L), L-arginine (503.50 mg/L), L-glutamine (505.30 mg/L), L-serine (78.50 mg/L), L-cysteine (13.10 mg/L), L-glycine (18.30 mg/L), L-methionine (31.40 mg/L), L-alanine (145.30 mg/L), L-threonine (75.92 mg/L), L-leucine (48.4 mg/L), L-valine (44.50 mg/L), L-phenylalanine (37.90 mg/L), L-isoleucine (32.70 mg/L), L-tyrosine (18.30 mg/L), L-proline (612.60 mg/L), L-tryptophan (179.30 mg/L), asparagine (5 mg/L), adapted from [23,24]. The final medium contained 1522.16 mg N/L and a pH of 4.3.

Subsequently, 200 μL of the mixture was transferred to the wells of a sterile 96-well microplate. The cell growth kinetics of each strain were assessed by recording absorbance every 30 min using a TECAN microplate reader. Absorbance measurements were taken from the microplates (OD_600_ nm × time) with agitation at 120 (r.p.m) before each reading in biological triplicate and technical triplicate. The experiments were carried out for 12–15 days at 28 °C in aerobic conditions.

### 2.3. Amino Acid Quantification

Amino acid concentrations were determined after derivatization with diethyl ethoxymethylenemalonate (DEEMM), following the method described by Rebane et al. [25]. The derivatized compounds were subsequently separated by HPLC using a Genesis Inertsil ODS-3 (250 × 4.6 mm) column coupled to a Shimadzu Prominence HPLC system (Shimadzu, Columbia, MD, USA). The gradient flow was Phase A: buffer sodium acetate 0.14 M, pH 5; and Phase B: acetonitrile:water 60:40. The separation gradient used was 0 min: 0% B; 13 min: 10% B; 19 min: 20% B; 26 min: 20% B; 29 min: 30% B; 34 min: 60%; 40 min: 60% B; 45 min: 100% B; 45.2 min: 0% B; 50 min: 0% B. Total run time was 50 min. The solvent flow was at 1.0 mL/min. The column temperature was maintained at 40 °C. The estimation of amino acids was undertaken using a diode array detector at a wavelength of 280 nm. The linearity range was between 0.1 and 100 mg/L. More details can be found in Supplementary Material S1.

For amino acid quantification, each strain was inoculated at an initial cell density of 1 × 10^7^ cells/mL (250 μL) into 25 mL of the YNB medium supplemented with 20 amino acids (YNB + 20 AAs), as described in Section 2.2. LAMAP1359 and LAMAP2480 exhibited broadly comparable growth kinetics under this condition; fixed sampling times of 48 and 72 h were selected for the initial comparative analysis. These sampling times were used exclusively for the assay supplemented with 20 AAs.

The residual concentration of each amino acid at 48 and 72 h was compared with its corresponding initial concentration at 0 h (Supplementary Table S1). Considering that a reduction in the concentration of amino acids in the culture medium (extracellular residual amino acid) cannot necessarily demonstrate its use as a nitrogen source, the term “apparent amino acid depletion” will be used in this work. The apparent decrease in each amino acid was normalized to the OD_600_ value measured at the corresponding sampling time and expressed as (mg/L)/OD_600_ (OD-AA depletion).

To evaluate the apparent decrease in amino acids as a function of nitrogen equivalents and OD_600_ (OD-AA-N depletion), the following metric was used, excluding nitrogen derived from ammonium sulfate.

OD-AA-N depletion t = [Σ_i_ ((C_i,0_ − C_i,t_) × ((n_i_ × 14.007)/MW_i_)]/OD_600,t_, where C_i,0_ and C_i,t_ are the initial and residual concentrations of amino acid i, respectively (mg/L); n_i_ is the number of nitrogen atoms in amino acid i; MW_i_ is the molecular weight of amino acid (g/mol); and OD_600,t_ is the optical density measured at the corresponding sampling time. The summation included all 20 amino acids quantified by HPLC, including proline. OD-AA–N depletion was expressed as ((mg N/L)/OD_600_).

### 2.4. Growth Kinetics of B. bruxellensis Strains Using a Single Amino Acid

A density of 1 × 10^7^ cells/mL (20 μL) was inoculated in 2 mL of yeast nitrogen base medium without amino acids (YNB), supplemented with 20 g/L of glucose, (ammonium sulfate (5 g/L) as the inorganic nitrogen source, along with biotin (2 μg/L), calcium pantothenate (40 μg/L), folic acid (2 μg/L), inositol (2 mg/L), nicotinic acid (400 μg/L), *p*-aminobenzoic acid (200 μg/L), pyridoxine HCl (400 μg/L), riboflavine (200 μg/L), thiamine HCl (400 μg/L), boric acid (500 μg/L), copper sulfate (40 μg/L), potassium iodide (100 μg/L), ferric chloride (200 μg/L), manganese sulfate (400 μg/L), sodium molybdate (200 μg/L), zinc sulfate (400 μg/L), monobasic potassium phosphate (1 g/L), magnesium sulfate (0.5 g/L), sodium chloride (0.1 g/L), and calcium chloride (0.1 g/L)) (Difco, Detroit, MI, USA), individually supplemented with one of the four amino acids that exhibited the highest apparent amino acid depletion (OD-AA depletion). The concentration of amino acids used separately for each condition was: L-arginine (503.50 mg/L), L-glutamine (505.30 mg/L), L-proline (612.60 mg/L), and L-tryptophan (179.30 mg/L). Growth curves were made using 96-well microplates with a final volume of 200 μL per well. OD_600_ was monitored every 30 min in a TECAN microplate reader (M200 Infinite Pro, TECAN, Männedorf, Switzerland). Absorbance measurements were taken from the microplates (OD_600_ nm × time) in aerobic conditions, with agitation at 120 (r.p.m) before each reading, in biological triplicate and technical triplicate. The experiments were carried out for 15 days at 28 °C in aerobic conditions.

### 2.5. Bioinformatic Analysis

In order to evaluate the presence of possible involved permeases in the transportation of amino acids found in the *B. bruxellensis* genome, sequences of three permeases were identified, using *S. cerevisiae* as model microorganism. For this, nucleotide sequences of genes of interest, namely, *GAP1*, *GNP1*, and *TAT1 S. cerevisiae* S288c, were obtained from the database KEGG (https://www.genome.jp/kegg/, accessed on 17 February 2026) of the Bioinformatics Center, Institute for Chemical Research, Kyoto University. The *B. bruxellensis* genome, LAMAP2480 strain, was obtained from the National Center for Biotechnology Information (NCBI) database (http://www.ncbi.nlm.nih.gov, accessed on 17 February 2026). In order to determine a sequence of interest related to the permeases involved in amino acids transportation, BLASTn (https://blast.ncbi.nlm.nih.gov/Blast.cgi?PROGRAM=blastn&BLAST_SPEC=GeoBlast&PAGE_TYPE=BlastSearch, accessed on 17 February 2026) local alignments were made between the nucleotide sequences of *S. cerevisiae* and *B. bruxellensis* LAMAP2480. NCBI BLASTn searches identified the putative homologs of *GAP1*, *GNP1*, and *TAT1*. The best hits showed 58% identity with 95% query coverage for *GAP1*, 48% identity with 78% query coverage for *GNP1*, and 39% identity with 93% query coverage for *TAT1*.

### 2.6. Gene Expression Assays

#### 2.6.1. RNA Extraction

For gene expression analysis, cells from single-amino-acid conditions were harvested when each culture reached OD_600_ equal to 0.2. Therefore, qPCR sampling was standardized by biomass/physiological growth level rather than by fixed incubation time, because growth kinetics differed markedly among strains and nitrogen source conditions. Three biological replicates and three technical replicates per strain and condition were processed. Total RNA was extracted according to Godoy et al. [26]. The complete RNA was purified using the RNA Clean & Concentrator Column (Zymo Research, Orange, CA, USA). It was quantified in the device Epoch (Biotek, Winooski, VT, USA) and then displayed through electrophoresis into agarose gel at 1% with ethidium bromide dye.

#### 2.6.2. qPCR Assays

cDNA was synthesized from protocol M-MLV Reverse Transcriptase (200 U/μL) (Invitrogen, Carlsbad, CA, USA), with 1 μg of total RNA as a template. q-PCR assays were made with StepOnePlus Real-Time PCR System Thermal Cycling Block equipment (Thermo Fisher Scientific, Waltham, MA, USA) using StepOne software (v2.0; Applied Biosystems, Foster City, CA, USA).

The primer sequences were as follows: (1) *GAP1*, F: GGTCAATCGAGTCCACAGGG, R: GGGATCCTGGTTTGGTCTCG (203 bp); (2) *GNP1*, F: GGTGGCCCTGCATCTGTAAT, R: GCCTGCAACAGGAAATGCAA (105 bp); and (3) *TAT1*, F: ACCACCTACTGCAAGAGCAC, R: AGAGCTCCACTTCAGCGAAA (114 bp), *ACT1*, F: GGTGATGACGCTCCAAGA, and R: TTGACCCATACCGACCATAA (120 bp)

qPCR reactions were performed in a final volume of 20 μL using 5× HOT FIREPol EvaGreen qPCR Mix Plus (ROX) (Solis Biodyne, Tartu, Estonia) according to the manufacturer’s instructions. The program used was: 95 °C for 15 min, followed by 40 cycles of 95 °C for 15 s, 50 °C for 15 s, and 72 °C for 15 s.

Each reaction was performed in technical and biological triplicate for each gene under study, using *ACT1* as a reference gene (housekeeping) [27]. Relative quantification of the genes of interest was performed using the 2^−ΔΔCT^ method described by Livak and Schmittgen [28]. To determine the efficiency (E) of the reaction and the correlation coefficient (R^2^) for the amplifications performed by each gene under study, the linear regression model estimated by Svec et al. [29] was used (Supplementary Table S2).

### 2.7. Statistical Analyses

All experiments were performed using three biological replicates, each measured in triplicate. Data were analyzed by one-way ANOVA followed by Fisher’s least significant difference (LSD) test using Statgraphics Plus v.18 (Manugistics, Inc., Rockville, VA, USA). Graphics and heatmaps were made using R v.4.3.3 and GraphPad Prism v.8 (GraphPad Software, San Diego, CA, USA).

## 3. Results

### 3.1. Strain-Dependent OD-AA Depletion in B. bruxellensis

**Figure 1:**
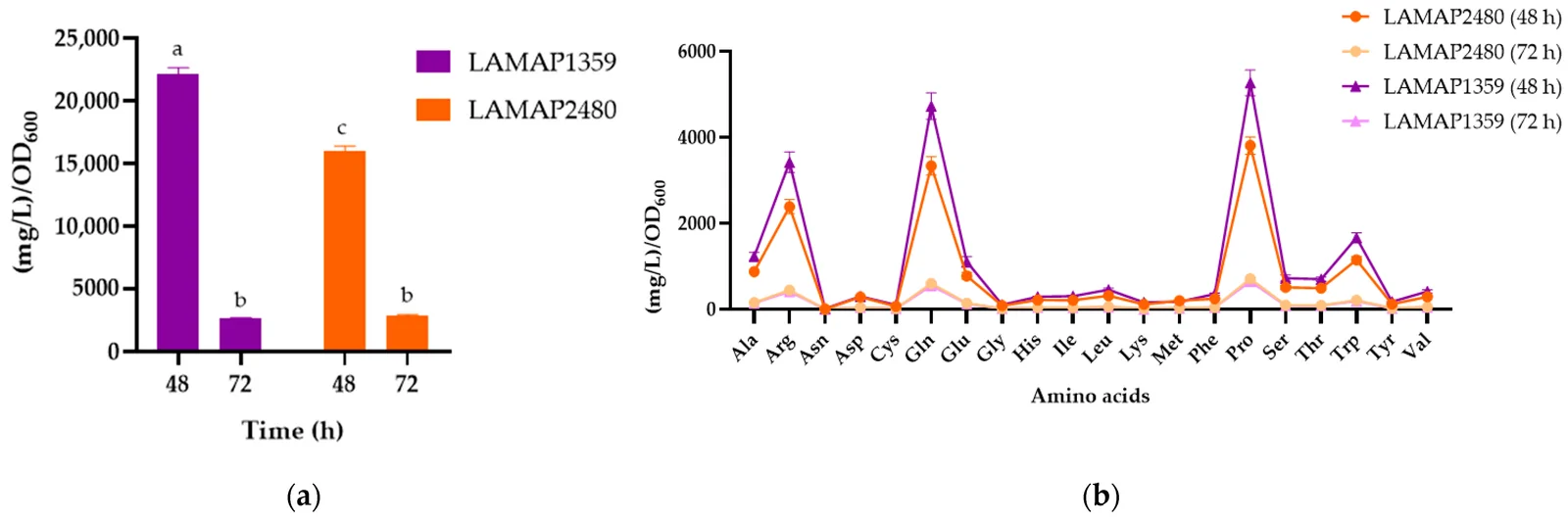
Apparent depletion of amino acids normalized to OD_600_ in *B. bruxellensis* strains. (**a**) Cumulative OD-AA depletion at 48 and 72 h. (**b**) OD-AA depletion of each amino acid quantified for LAMAP1359 and LAMAP2480 at 48 and 72 h. All experiments were performed with three biological replicates, each measured in triplicate. Different letters indicate statistically significant differences between strains at the different time. Statistical differences were determined by one-way ANOVA followed by Fisher’s LSD (*p* < 0.05).

In this research, we aimed to determine whether apparent amino acid depletion differed between *B. bruxellensis* strains, and whether these differences were associated with strain-dependent growth behavior. For this, two *B. bruxellensis* strains were grown in a supplemented medium with 20 amino acids (YNB + 20AAs) observing their effect on 48 h and 72 h (Figure 1, Table 1).

Regarding within-strain patterns, both strains exhibited a similar OD-AA depletion hierarchy at 48 and 72 h. Proline showed the highest depletion, followed by glutamine, arginine, tryptophan, alanine, and glutamic acid, whereas serine and threonine displayed intermediate values. The lowest values were generally observed for asparagine, cysteine, glycine, and lysine, with lysine being the least depleted amino acid in LAMAP1359 and cysteine being the least depleted amino acid in LAMAP2480 at 72 h. Overall, the relative depletion profile remained largely conserved between strains and sampling times. In the case of OD-AA depletion between strains at the different sampling times, no significant differences were observed for aspartic acid, glycine, or methionine at 48 h, whereas all other amino acids exhibited significant strain-dependent differences. At 72 h, significant differences between strains were detected only for asparagine, glycine, lysine, and methionine, while no significant differences were observed for the remaining amino acids.

**Figure 2:**
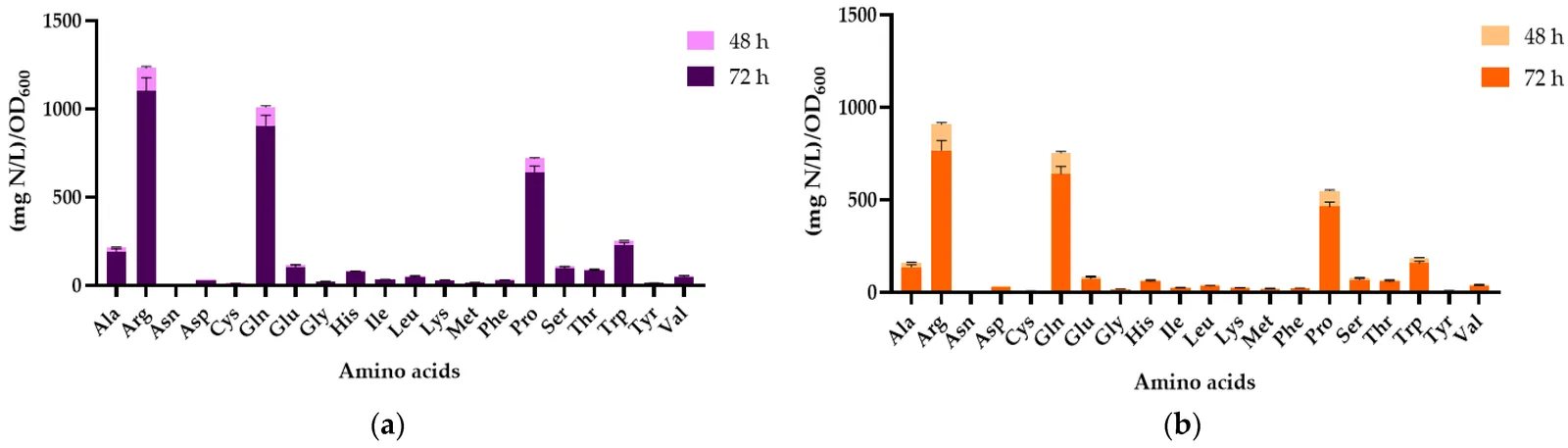
OD-AA-N depletion of two *B. bruxellensis* strains. All experiments were performed with three replicates, each measured in triplicate: (**a**) OD-AA-N depletion at 48 and 72 h for LAMAP1359; (**b**) OD-AA-N depletion at 48 and 72 h for LAMAP2480.

For this investigation, we used a comparative physiological index of amino acid-derived nitrogen (OD-AA-N depletion) for each strain under the defined aerobic laboratory conditions (Figure 2).

Under the assayed conditions, LAMAP1359 showed an OD-AA-N depletion of 3710.14 (mg N/L)/OD_600_), whereas LAMAP2480 showed an OD-AA-N depletion of 2632.35 (mg N/L)/OD_600_).

**Figure 3:**
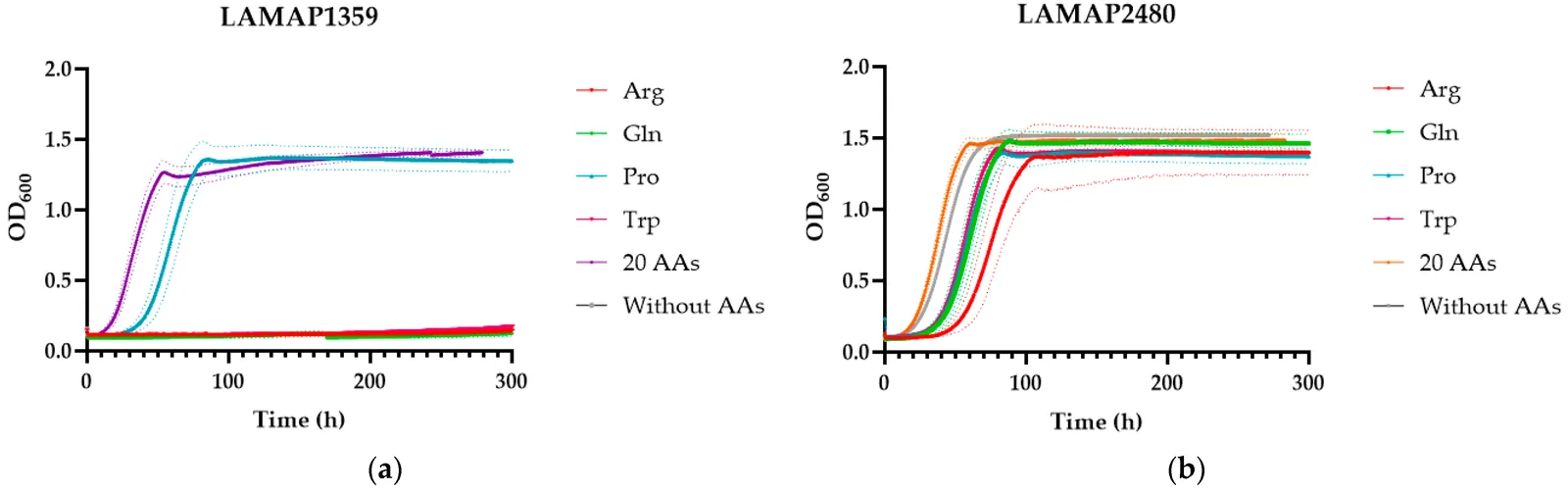
Growth kinetics of *B. bruxellensis* evaluated over 300 h. Experiments were performed with three replicates, each measured in triplicate. Solid lines represent mean growth and dotted lines represent ± SD. (**a**) *B. bruxellensis* LAMAP1359. (**b**) *B. bruxellensis* LAMAP2480. YNB + 20AAs: purple; YNB medium independently supplemented with arginine (503.50 mg/L): red; glutamine (505.30 mg/L): green; proline (612.60 mg/L): light blue; tryptophan (179.30 mg/L): pink; YNB with ammonium sulfate without added amino acids: gray.

To determine the behavior of the strains selected in this study, the growth kinetics of both strains were evaluated in a culture medium supplemented with 20Aas (Figure 3, Table 2).

Under the YNB + 20AAs condition, both strains showed broadly comparable lag phases and reached high final OD_600 nm_ values, supporting the use of fixed time points as an initial comparative strategy for amino acid quantification. However, when cultures were supplemented with a single added amino acid in the presence of ammonium sulfate, the kinetic behavior differed markedly between strains. LAMAP1359 showed the greatest variation in kinetic parameters, with strongly prolonged lag phases, increased generation times, and lower final biomass in single-amino-acid and no-added-amino-acid conditions. Compared with the YNB + 20AAs condition, the generation time of LAMAP1359 increased approximately 16.3-, 15.0-, 11.0-, and 9.3-fold in the presence of glutamine, tryptophan, arginine, and proline, respectively. In contrast, LAMAP2480 maintained high final biomass across conditions, although single amino acid supplementation increased lag time relative to YNB + 20AAs. These results indicate that amino acid availability shapes strain-dependent physiology in *B. bruxellensis*, particularly under conditions with limited added organic nitrogen. Moreover, these comparisons indicate that the behavior of *B. bruxellensis* depends on the studied strain [14].

### 3.2. Gene Expression Assays

Growth kinetics in ammonium-containing YNB individually supplemented with arginine, glutamine, proline, or tryptophan revealed strain- and amino acid-dependent responses. LAMAP1359 exhibited marked growth in the presence of proline but limited growth with the other three amino acids, whereas LAMAP2480 grew under all four conditions, although with differences in lag phase and the OD_600_ reached. Based on these phenotypes, the putative homologs *GAP1*, *GNP1*, and *TAT1* were selected to determine whether the observed growth differences were accompanied by distinct transcriptional responses associated with amino acid transport. *GAP1* was selected as a candidate for the arginine- and proline-supplemented conditions because, in *S. cerevisiae*, it encodes a broad specificity permease capable of transporting various amino acids [30]. *GNP1* was selected because it encodes a high-affinity glutamine permease [31], and this amino acid has also been associated with NCR regulation in *B. bruxellensis* [19]. Finally, *TAT1* was analyzed due to its contribution to tryptophan uptake [32]. Taken together, these results allowed for the evaluation of a general transport response associated with arginine, proline, glutamine, and tryptophan. However, substrate specificity cannot be inferred for the proteins encoded by these putative genes in *B. bruxellensis*.

**Figure 4:**
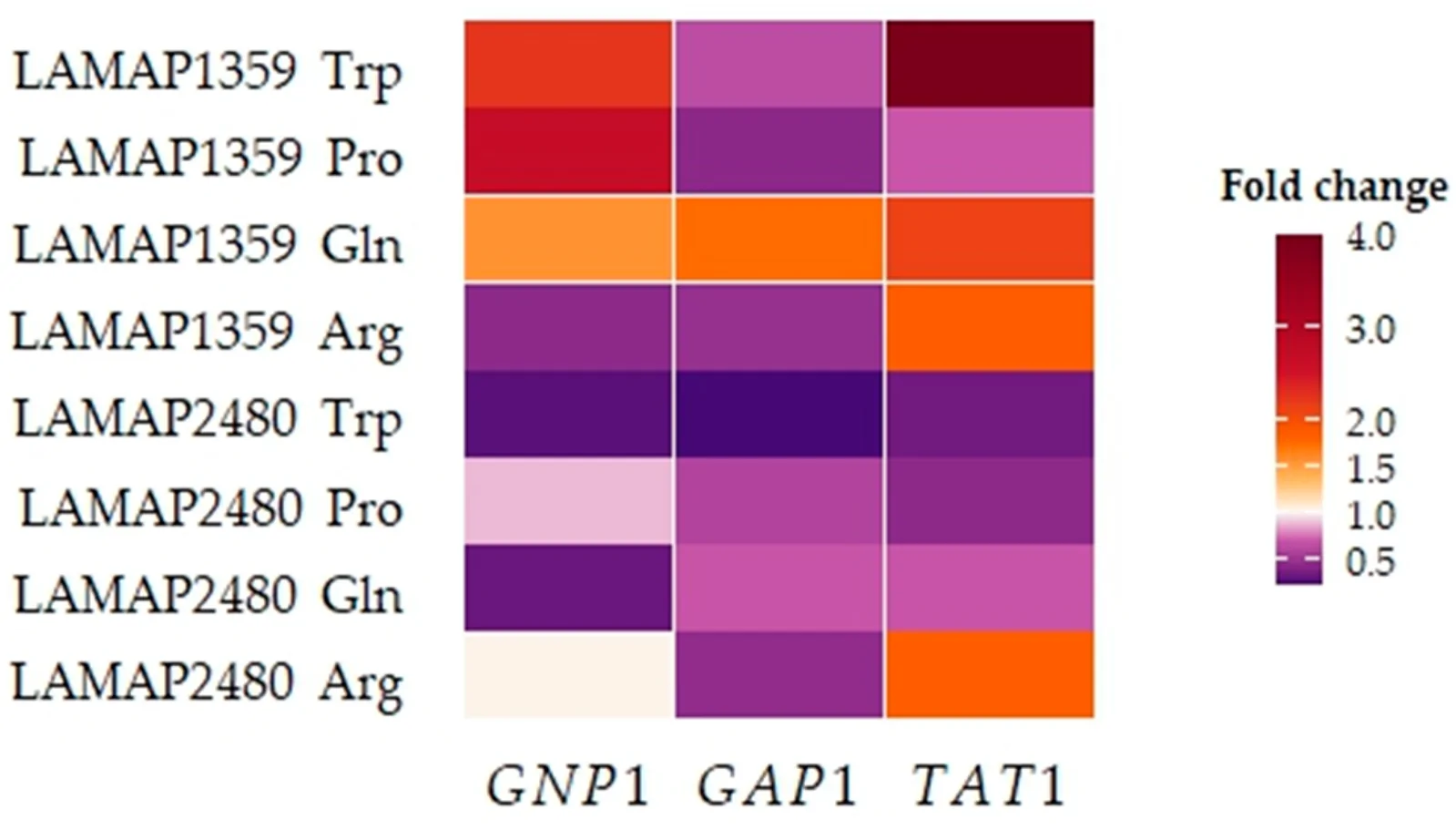
Heat map of the relative gene expression of *GAP1*, *GNP1*, and *TAT1* in LAMAP1359 and LAMAP2480 strains grown in a medium supplemented with only one of the four selected amino acids (arginine, proline, tryptophan, or glutamine) at an OD_600_ of 0.2. Values less than 1 indicate a lower relative abundance of transcripts compared to the YNB + 20 AAs reference condition; values greater than 1 indicate a higher relative abundance of transcripts. All experiments were performed with three biological replicates, each measured in triplicate.

Expression of each gene was evaluated using qPCR assays when yeasts were grown separately in a YNB medium supplemented with 20 amino acids (YNB + 20AAs) and a YNB medium supplemented with one of the four selected amino acids (glutamine, arginine, proline, and tryptophan) (Figure 4, Supplementary Table S3).

Both strains showed distinct relative transcript abundance profiles for the evaluated genes. For *GAP1*, only LAMAP1359 showed higher relative transcript abundance when grown in the medium supplemented with glutamine as the single added amino acid, with a 1.75-fold increase relative to the YNB + 20 AAs condition. For *GNP1*, LAMAP1359 showed higher relative transcript abundance in the presence of tryptophan, proline, and glutamine, with 2.24-, 2.69-, and 1.56-fold increases, respectively. For *TAT1*, LAMAP1359 showed higher relative transcript abundance in the presence of tryptophan, glutamine, and arginine, with 3.96-, 2.12-, and 1.86-fold increases, respectively. In contrast, the LAMAP2480 strain showed higher relative transcript abundance for *TAT1* in the presence of arginine, with a 1.84-fold increase. These results only suggest strain- and nitrogen source-dependent transcriptional regulation in the two strains analyzed.

## 4. Discussion

Fungal nitrogen metabolism plays a fundamental role in yeast growth, adaptation, and survival in fermentation environments. In this study, comparative analysis of two strains of *B. bruxellensis* revealed that amino acid depletion is highly strain-dependent and subject to varying nitrogen availability conditions. Several studies have demonstrated the diversity in nitrogen consumption among yeast strains, as well as the variability in the assimilation rate of nitrogenous compounds [23,33]. *S. cerevisiae* has been described as preferring some nitrogen sources, such as ammonium, glutamate, and aspartate, while urea and proline are not preferred [34,35]. Previous studies aimed at characterizing nitrogen source utilization in *B. bruxellensis* have reported that this yeast can assimilate various amino acids, including proline and arginine, as nitrogen sources; however, the degree of amino acid utilization appears to depend on the strain and conditions [15,17].

Parente et al. [18] evaluated the growth kinetics of the sexually reproducing strain *Dekkera bruxellensis* GDB 248 in culture media containing glucose as the carbon and nitrogen source. Glutamine was the preferred nitrogen source, regardless of oxygen availability, likely due to its central role as the main intracellular nitrogen sensor in this yeast strain [19]. Consistent with that report, glutamine showed high apparent depletion under the aerobic conditions in the two study strains.

Regarding other amino acids, Niu et al. [36] demonstrated in *S. pastorianus* that intracellular proline accumulation may be important for resistance under unfavorable conditions, such as high sugar or ethanol concentrations and osmotic stress, and that it could also serve as an energy source for the yeast. *B. bruxellensis* LAMAP1359 and LAMAP2480 strains showed the greatest apparent depletion of four amino acids at 48 and 72 h under the tested conditions (arginine, glutamine, tryptophan, and proline) (Figure 1). Although proline is the most abundant amino acid in grape must, *Saccharomyces* spp. does not completely consume it during alcoholic fermentation. This can lead to negative sensory problems, such as increased bitterness, decreased acidity, and a milky appearance in the final product [37]. Arginine in the medium has been described as inhibiting proline consumption in most *S. cerevisiae* strains [38]. However, proline is consumed by non-*Saccharomyces* yeasts such as *Metschnikowia* spp., *Millerozyma* spp., *Papiliotrema* spp., *Rhodotorula* spp., *Kazachstania* spp., and *Wickerhamomyces* spp. [37]. Our study showed that proline was the most depleted in both strains evaluated, with 5266.38 ± 296.49 and 3806.85 ± 202.62 (mg/L/OD_600_), respectively (Figure 1, Table 1). However, when proline was the sole added amino acid in ammonium-containing YNB, growth responses differed markedly between strains, indicating that its contribution depended on both strain and nutritional context. Mukai et al. [39] reported that the high metabolism of proline plays a physiological role in maintaining the viability of yeast cells. Therefore, *B. bruxellensis* could represent a suitable example of a microorganism exposed to selective pressures from nutrient-limited environments [13,14].

Although our results highlight strain-dependent differences in apparent amino acid depletion and growth responses, these physiological patterns must be interpreted cautiously, considering the interactive regulation of carbon and nitrogen metabolism. In high-glucose media, amino acid utilization may be significantly modulated by carbon catabolite repression [9]. As amino acids can also serve as alternative carbon sources, the growth difference observed using OD_600_ measurements could arise from variations in carbon metabolism and not only solely from nitrogen assimilation pathways—a point to consider for future research. Gobert et al. [40] reported that strain background, nitrogen source, cultivation temperature, and shaking conditions should be considered when evaluating nitrogen source preference.

This consideration is particularly relevant for the present study, because fixed sampling times are appropriate only when growth progression is broadly comparable between strains. In contrast, the single-amino-acid conditions produced highly divergent kinetic responses, especially in the LAMAP1359 strain, which showed extended lag phases and reduced final biomass. For this reason, gene expression analyses were standardized at OD_600_ 0.2 rather than at a fixed incubation time, allowing comparisons to be made at a comparable biomass/physiological growth level.

Molinet et al. [41] propose that the difference in nitrogen consumption is due to the different mechanisms regulating nitrogen assimilation at the genetic level. The order of consumption of nitrogen compounds is related to the expression of genes involved in the processes of incorporation and assimilation, which are generally controlled by internal regulatory mechanisms that ultimately choose the order of consumption [33]. This strategy allows cells to control the appropriate use of nitrogen sources and is the basis of NCR [7]. In our study, differences in transcriptional regulation were observed in putative genes related to nitrogen transport (Figure 4). These transcriptional responses were strain-specific and dependent on the nitrogen sources.

Since NCR genes, such as *GLN3*, *GAT1*, *URE2*, and *DAL80/GZF3*, were not evaluated, this study does not provide evidence of an NCR-like regulation in *B. bruxellensis*. Our result provides preliminary evidence that the expression of selected amino acid permease genes is modulated by the strain’s genetic background and the nitrogen source composition. Cajueiro et al. [19] investigated NCR-related gene expression in *B. bruxellensis* and suggested that intracellular glutamine availability may contribute to the regulation of NCR-responsive genes. The *B. bruxellensis* LAMAP2480 genome sequence contains several genes associated with nitrogen source utilization, supporting the relevance of nitrogen metabolism in this organism [42].

The strain-dependent physiological and transcriptional differences observed here are consistent with the high genomic and phenotypic diversity described for *B. bruxellensis*. However, since the ploidy level and acquired subgenome content of the LAMAP1359 and LAMAP2480 strains were not directly assessed in this study, these genomic characteristics should be considered a possible explanatory framework rather than a proven causal mechanism.

In *S. cerevisiae*, amino acid uptake involves multiple permeases, including Gap1p, the general amino acid permease; Agp1p, a broad substrate permease; and Put4p, the proline-specific permease. Their expression and activity are regulated by nitrogen availability. Lleixà et al. [8] evaluated the gene expression patterns of these permeases in *S. cerevisiae* and *Hanseniaspora vineae*, both grown in synthetic must supplemented with 20 amino acids as a nitrogen source. The results showed that *GAP1* was repressed in both strains. However, after 48 h, an increase in the expression of this permease was observed. This behavior was similar to that of one of the *B. bruxellensis* strains studied, since the expression of *GAP1* was higher than that of the other permeases evaluated. The amino acid permease Gap1p is activated when the microorganism grows in the presence of a non-preferred nitrogen source and is repressed in the presence of a preferred nitrogen source such as ammonium [43,44]. Furthermore, other non-*Saccharomyces* yeasts, such as *Torulaspora delbrueckii* and *Lanchancea thermotolerans*, assimilate amino acids through a mechanism similar to NCR [45].

Therefore, integrating growth kinetics, amino acid utilization, and the expression of selected permease genes provides a useful framework for understanding fungal nitrogen metabolism and strain-dependent adaptation in *B. bruxellensis*. Finally, the novelty of this work lies in its combined evaluation of physiological performance, amino acid consumption, apparent nitrogen depletion derived from amino acids normalized to OD_600_ (OD-AA–N depletion), and the transcriptional responses of selected amino acid permease genes, which allows for the use of nitrogen sources in *B. bruxellensis* to be interpreted as a strain-dependent trait rather than as a fixed metabolic behavior.

*Study limitations*: The present study provides new evidence on the ability of *B. bruxellensis* to metabolize different amino acids as nitrogen sources. As parameters such as glucose consumption, ethanol production, and dry cell weight were not measured, growth differences arising from carbon metabolism cannot be ruled out. Although the analysis of the three permeases genes yielded preliminary insights, it is insufficient to definitively determine whether an NCR-like pathway was activated or repressed. Furthermore, it is necessary to conduct new experiments considering other regulatory genes associated with NCR. Likewise, more research under anaerobic conditions is required to link the studied conditions to a fermentation process. In addition, to fully characterize the yeast’s behavior in wine, additional factors must be evaluated, including pH, concentration of glucose, fructose, ethanol, and sulfur dioxide, as well as the presence of competing microorganisms.

## 5. Conclusions

Nitrogen consumption is essential for yeast growth, adaptation, and persistence in fermentative environments. The marked differences observed between LAMAP1359 and LAMAP2480 strains during growth on culture medium with ammonium sulphate supplemented with individual amino acids further support the existence of strain and nitrogen source dependence. However, this condition should be interpreted as limited in added organic nitrogen rather than completely nitrogen free, because the contribution of ammonium sulfate or residual nitrogen sources must be considered. In addition, differences in the relative expression of selected putative amino acid permease genes indicate that nitrogen source availability may be associated with strain-dependent transcriptional responses related to amino acid transport. These results provide preliminary evidence that amino acid depletion and permease expression are associated with the physiological adaptation of this yeast. Overall, this study highlights amino acid utilization as a strain-dependent trait in *B. bruxellensis* and supports the relevance of nitrogen metabolism as a potential factor contributing to its survival in nutrient-limited fermented environments. Future studies are necessary to demonstrate that the nitrogen source is incorporated into cellular metabolism. Likewise, it is necessary to evaluate other factors that can influence the nitrogen metabolism, such as survival under nitrogen limitation and fermentative conditions, competition with other microorganisms, ethanol tolerance, etc. This will help us to understand how *B. bruxellensis* manages to survive in ecosystems as unfavorable as wine.

## Figures and Tables

**Table 1:** Biomass-normalized apparent depletion of individual amino acids by *B. bruxellensis* LAMAP1359 and LAMAP2480 in ammonium-containing YNB supplemented with 20 amino acids. Values are expressed as (mg/L)/OD_600_. All experiments were performed using three biological replicates, each measured in triplicate. Different lowercase letters within the same column indicate statistically significant differences among apparent amino acid depletion for a given strain. Different uppercase letters within the same row indicate statistically significant differences between strains for each apparent amino acid depletion at the same sampling time. Statistical differences were determined by one-way ANOVA followed by Fisher’s LSD (*p* < 0.05).

Amino Acids	LAMAP1359 48 h (mg/L)/OD_600_	LAMAP2480 48 h (mg/L)/OD_600_	LAMAP1359 72 h (mg/L)/OD_600_	LAMAP2480 72 h (mg/L)/OD_600_
Ala	1226.75 ± 96.49 ^(h,A)^	871.71 ± 68.23 ^(I,B)^	143.70 ± 10.92 ^(h,A)^	154.30 ± 11.25 ^(h,A)^
Arg	3419.90 ± 237.07 ^(j,A)^	2382.81 ± 167.44 ^(k,B)^	408.74 ± 28.15 ^(j,A)^	440.23 ± 30.56 ^(j,A)^
Asn	11.81 ± 1.09 ^(a,A)^	2.30 ± 0.13 ^(a,B)^	1.03 ± 0.18 ^(a,b,A)^	0.29 ± 0.01 ^(a,b,B)^
Asp	296.32 ± 14.2 ^(a,b,c,d,e,A)^	280.62 ± 6.62 ^(b,c,d,e,f,A)^	43.75 ± 2.51 ^(c,d,e,f,A)^	40.81 ± 1.07 ^(a,b,c,d,e,A)^
Cys	99.27 ± 8.51 ^(a,A)^	69.28 ± 5.20 ^(a,B)^	13.25 ± 0.92 ^(a,b,A)^	13.86 ± 0.97 ^(a,A)^
Gln	4724.55± 307.98 ^(k,A)^	3333.01 ± 216.72 ^(l,B)^	549.20 ± 35.72 ^(k,A)^	597.92 ± 38.87 ^(k,A)^
Glu	1101.19 ± 118.51 ^(g,A)^	773.60 ± 82.90 ^(h,B)^	126.22 ± 13.53 ^(g,A)^	137.94 ± 14.84 ^(g,A)^
Gly	102.72 ± 12.50 ^(a,A)^	80.72 ± 8.92 ^(a,A)^	10.88 ± 1.05 ^(a,b,B)^	14.98 ± 1.14 ^(a,b,A)^
His	285.71 ± 15.79 ^(b,c,A)^	213.11 ± 11.85 ^(d,e,B)^	35.06 ± 1.95 ^(d,e,A)^	37.68 ± 2.08 ^(c,d,A)^
Ile	301.09 ± 18.29 ^(c,d,A)^	207.68 ± 12.14 ^(d,e,B)^	33.48 ± 2.07 ^(c,d,e,A)^	37.12 ± 2.29 ^(c,d,A)^
Leu	453.19 ± 35.98 ^(e,A)^	313.74 ± 25.08 ^(f,B)^	51.56 ± 4.12 ^(f,A)^	56.40 ± 4.51 ^(e,A)^
Lys	156.58 ± 11.87 ^(a,A)^	110.78 ± 8.38 ^(a,b,B)^	0.07 ± 0.02 ^(a,B)^	19.80 ± 1.50 ^(a,b,A)^
Met	174.22 ± 17.84 ^(a,b,A)^	197.03 ± 14.50 ^(c,d,A)^	21.64 ± 1.99 ^(b,c,d,B)^	29.96 ± 1.98 ^(b,c,A)^
Phe	354.25 ± 24.20 ^(c,d,e,A)^	244.53 ± 16.73 ^(d,e,f,B)^	40.14 ± 2.68 ^(e,f,A)^	43.85 ± 3.03 ^(c,d,e,A)^
Pro	5266.38 ± 296.49 ^(l,A)^	3806.85± 202.62 ^(m,B)^	649.71 ± 34.31 ^(l,A)^	706.05 ± 37.30 ^(l,A)^
Ser	723.93 ± 68.67 ^(f,A)^	506.58 ± 48.18 ^(g,B)^	83.16 ± 7.87 ^(f,A)^	91.11 ± 8.64 ^(f,A)^
Thr	698.76 ± 48.65 ^(f,A)^	488.02 ± 34.07 ^(g,B)^	80.10 ± 5.58 ^(f,A)^	87.36 ± 6.07 ^(f,A)^
Trp	1661.76 ± 114.69 ^(I,A)^	1146.32 ± 79.27 ^(j,B)^	189.25 ± 13.11 ^(I,A)^	206.50 ± 14.40 ^(I,A)^
Tyr	170.67 ± 9.97 ^(a,b,A)^	118.70 ± 6.93 ^(a,b,c,B)^	19.42 ± 1.14 ^(b,c,A)^	21.44 ± 1.24 ^(a,b,A)^
Val	413.47 ± 41.13 ^(d,e,A)^	288.90 ± 28.73 ^(e,f,B)^	47.32 ± 4.71 ^(e,f,A)^	51.68 ± 5.14 ^(d,e,A)^

**Table 2:** Kinetic parameters after 300 h of growth for *B. bruxellensis* strains. Differences were determined by one-way ANOVA followed by Fisher’s LSD (*p* < 0.05). Letters in the same column show statistically significant differences between conditions on the same strain. All experiments were performed using three biological replicates, each measured in triplicate.

Strain	Culture Medium	Specific Growth Rate µ (h^1^)	Generation Time (h)	Lag Phase (h)	Final OD (OD_600_)
LAMAP1359	YNB without AAs	0.003 ± 0.0006 ^(a)^	212.111 ± 31.66 ^(b)^	283.59 ± 3.58 ^(d)^	0.1075 ± 0.06 ^(a)^
LAMAP1359	YNB + 20AAs	0.039 ± 0.002 ^(b)^	17.404 ± 0.909 ^(a)^	14.50 ± 1.38 ^(a)^	1.073 ± 0.09 ^(c)^
LAMAP1359	YNB + Trp	0.002 ± 0.0004 ^(a)^	261.175 ± 41.234 ^(b)^	229.00 ± 1.25 ^(b)^	0.202 ± 0.027 ^(b)^
LAMAP1359	YNB + Pro	0.004 ± 0.0008 ^(a)^	162.281 ± 29.724 ^(b)^	230.00 ± 2.187 ^(b)^	0.226 ± 0.04 ^(b)^
LAMAP1359	YNB + Gln	0.002 ± 0.0003 ^(a)^	282.879 ± 34.578 ^(b)^	251.00 ± 1.50 ^(c)^	0.214 ± 0.017 ^(b)^
LAMAP1359	YNB + Arg	0.003 ± 0.0006 ^(a)^	191.062 ± 36.491 ^(b)^	264.50 ± 5.52 ^(c,d)^	0.310 ± 0.069 ^(b,c)^
LAMAP2480	YNB without AAs	0.061 ± 0.00005 ^(a)^	11.279 ± 0.015 ^(a)^	12.00 ± 1.06 ^(a)^	1.487 ± 0.03 ^(a)^
LAMAP2480	YNB + 20AAs	0.029 ± 0.001 ^(c)^	23.596 ± 1.115 ^(c)^	12.50 ± 0.88 ^(a)^	1.332 ± 0.054 ^(a)^
LAMAP2480	YNB + Trp	0.042 ± 0.0006 ^(b)^	16.323 ± 0.248 ^(b)^	26.00 ± 0.14 ^(b)^	1.392 ± 0.019 ^(a)^
LAMAP2480	YNB + Pro	0.040 ± 0.001 ^(b)^	17.217 ± 0.495 ^(b)^	29.50 ± 0.88 ^(b,c)^	1.344 ± 0.076 ^(a)^
LAMAP2480	YNB + Gln	0.038 ± 0.001 ^(b)^	17.946 ± 0.509 ^(b)^	25.50 ± 0.68^(b)^	1.460 ± 0.067 ^(a)^
LAMAP2480	YNB + Arg	0.033 ± 0.002 ^(b,c)^	20.887 ± 1.506 ^(a,b)^	33.00 ± 0.46 ^(c)^	1.397 ± 0.161 ^(a)^

## Data Availability

The datasets generated and analyzed during the current study are available from the corresponding author upon reasonable request.

## Supplementary Materials

The following supporting information can be downloaded at https://www.mdpi.com/article/10.3390/jof12090636/s1. Material S1: Chromatographic conditions for the determination of amino acids by HPLC; Table S1: Quantification of residual amino acids and percentage removed from the culture medium for *B. bruxellensis* LAMAP1359 and 2480 strains. Table S2: Efficiency (E) of the reaction and correlation coefficient (R^2^) and slope for the amplifications performed by each gene under study. Table S3: Relative expression of amino acid permease genes in *B. bruxellensis* LAMAP2480 and LAMAP1359 strains under different amino acid conditions.
