# Supplementary material for: Amino Acid Depletion Reveals Strain- and Nitrogen Source-Dependent Physiological Responses in Brettanomyces bruxellensis
Source: J Fungi (Basel). 2026 Aug 26;12(9):636. doi: 10.3390/jof12090636 (PMC13608304; doi:10.3390/jof12090636)
Supplement: Supplementary file 1 [file jof-12-00636-s001.zip › jof-4464171-supplementary.pdf]

Material S1: Chromatographic conditions for the determination of amino acids by HPLC

Equipment	HPLC Shimadzu model Prominence
Methods	Rebane, R. and Herodes, K. (2010). A sensitive method for free amino acids analysis by liquid chromatography with ultraviolet and mass spectrometric detection using precolumn derivatization with diethyl ethoxymethylenemalonate: Application to the honey analysis. Anal. Chim. Acta 672: 79–84. https://doi.org/10.1016/j.aca.2010.04.014
Software	LC Solution
Columna	Inertsil ODS-3 (250 x 4.6 mm)
Rate flow	1.0 mL/min
Gradient flow	Phase A: Buffer sodium acetate 0.14M pH 5.8<br>Phase B: Acetonitrile: water 60:40

Gradient conditions:

Time	Flow	%Phase A	% Phase B
0	1.0	100	0
13	1.0	90	10
19	1.0	80	20
26	1.0	80	20
29	1.0	70	30
34	1.0	40	60
40	1.0	40	60
45	1.0	0	100
45.2	1.0	100	0
50	1.0	100	0

Standard used	external standard
Over temperature	40º C
Detector	Arreglo de diodos (SPD- M20A).
Wavelength	280nm
Derivatization reactive	Dietiletoximetilmalonato (DEEM)
Linearity range	0.1-100 mg/L

Characteristic of HPLC technique used for amino acid analysis

Amino acids	Standard deviation	LOD (mg/L)	LOQ (mg/L)	Recovery (%)	Percentage Coefficient of Variation (%)
Asp	0.31	0.93	3.11	101.8	3.18
Glu	0.78	2.33	7.77	100.6	3.02
Ser	0.75	2.24	7.48	99.9	4.59
His	0.38	1.15	3.85	101.6	3.52
Gln	1.88	5.63	18.75	99.0	2.83
Gly	0.03	0.10	0.33	99.8	0.82
Arg	1.85	5.55	18.50	100.8	2.53
Thr	0.30	0.91	3.04	100.0	2.04
Ala	0.41	1.24	4.12	98.9	1.48
Pro	1.91	5.72	19.08	101.2	1.43
Tyr	0.13	0.38	1.26	101.4	3.25
Ammonium	1.96	5.87	19.56	101.3	1.95
Val	0.19	0.56	1.87	98.1	2.18
Met	0.02	0.06	0.19	101.4	1.43
Cys	0.20	0.59	1.98	100.9	1.73
Try	1.32	3.95	13.17	99.1	4.05
Iso	0.16	0.47	1.57	101.1	1.81
Leu	0.12	0.35	1.16	98.9	1.29
Phen	2.46	7.37	24.55	100.6	2.51
Lys	0.08	0.24	0.78	99.6	2.41

LOD: Limit of detection, LOQ: Limit of quantification

Table S1: Quantification of residual amino acids and percentage removed from the culture medium for *B. bruxellensis* LAMAP1359 and 2480 strains. All experiments were performed using three biological replicates, and each measured in triplicate.

	**Without inoculation (control - 0 h)**	**LAMAP1359 48 h**		**LAMAP2480 48 h**		**LAMAP1359 72 h**		**LAMAP2480 72 h**		**Without inoculation (control)**
**Amino acids**	Initial amino acid concentration in the culture medium (mg/L)	Residual amino acid concentration in the culture medium (mg/L)	Percentage removed (%)	Residual amino acid concentration in the culture medium (mg/L)	Percentage removed (%)	Residual amino acid concentration in the culture medium (mg/L)	Percentage removed (%)	Residual amino acid concentration in the culture medium (mg/L)	Percentage removed (%)	Final amino acid concentration in the culture medium (mg/L)
Ala	154.21±5.75	15.01± 0.51	90.27	14.27±1.26	90.75	14.10±0.51	90.86	16.30±2.30	89.43	147.48±12.66
Arg	437.22±46.59	15.37±0.44	96.48	16.42±1.39	96.24	0.97±0.07	99.78	5.19±0.29	98.81	382.26±30.83
Cys	13.72±0.87	2.76±0.12	79.84	2.89±0.20	78.88	1.04±0.11	92.39	1.54±0.10	88.72	13.51±0.,72
Phe	39.62±1.10	0.31±0.006	99.21	0.82±0.08	97.93	0.98±0.09	97.52	0.81±0.06	97.94	39.04±2.55
Gly	18.97±0.43	7.23±0.63	61.89	6.07±0.73	67.99	8.57±0.37	54.83	5.69±0.32	70.01	17.57±1.56
Glu	127.17±8.35	3.52±0.06	97.23	3.03±0.34	97.62	4.11±0.19	96.77	3.69±0.28	97.10	127.39±8.25
Gln	522.12±10.27	5.17±0.12	99.01	0.60±0.06	99.88	0.31±0.00	99.94	0.51±0.05	99.90	526.65±24.12
His	46.54±1.98	2.19±0.16	95.29	0.03±0.00	99.93	0.06±0.00	99.86	0.52±0.02	98.87	33.69±1.11
Ile	34.06±1.11	0.63±0.03	98.13	1.14±0.07	96.65	1.74±0.11	94.87	1.18±0.07	96.54	34.02±1.56
Leu	51.34±3.15	0.34±0.01	99.33	0.83±0.035	98.38	0.93±0.05	98.18	0.72±0.09	98.59	50.81±3.81
Lys	17.58±6.51	0.29±0.01	98.31	0.10±0.008	99.43	0.23±0.03	98.69	0.16±0.01	99.09	17.,32±1.23
Met	33.06±1.80	13.36±0.44	59.57	1.67±0.11	94.95	11.90±0.35	63.98	6.74±0.37	79.60	33.28±2.14
Pro	612.61±37.04	43.19±2.48	92.95	23.23±2,99	96.21	1.77±0.15	99.71	3.17±0.16	99.48	618.13±35.84
Ser	82.98±4.01	0.89±0.03	98.92	0.82±0,064	99.01	1.09±0.11	98.68	0.62±0.05	99.25	82.13±8.28
Tyr	19.05±0.61	0.13±0.005	99.31	0.23±0,015	98.75	0.35±0.05	98.12	0.10±0.008	99.45	19.01±1.06
Thr	77.77±3.21	0.11±0.005	99.86	0.21±0,016	99.73	0.47±0.04	99.39	0.36±0.01	99.53	76.24±4.53
Trp	186.38±10.35	2.91±0.15	98.44	5.40±0,26	97.10	5.06±0.34	97.28	4.67±0.60	97.49	186.63±12.05
Val	47.02±2.77	0.07±0.002	99.84	0.12±0,004	99.75	0.37±0.01	99.21	0.24±0.01	99.49	46.62±4.21
Asn	4.79±0.62	1.26±0.02	73.64	0.35±0,001	92.65	0.96±0.00	79.90	0.25±0.01	94.70	4.68±0.21
Asp	44.51±1.80	12.80±1.30	71.24	1.57±0,09	96.47	3.86±0.03	91.32	9.69±0.01	78.23	44.1±1.11
**DO_{600}**	-	0.107±0.01	-	0.153±0.02	-	0.929±0.02	-	0.853±0.05	-	-

Table S2: Efficiency (E) of the reaction and correlation coefficient (R^2) and slope for the amplifications performed by each gene under study.

Gen	Primers	Slope	Correlation coefficient (R^2)	Efficiency (%)
GNP1	F: GGTCAATCGAGTCCACAGGG<br>R: GGGATCCTGGTTTGGTCTCG	-3.678	0.985	87.0
GAP1	F: GGTGGCCCTGCATCTGTAAT<br>R: GCCTGCAACAGGAAATGCAA	-3.57	0.996	90.6
TAT1	F: ACCACCTACTGCAAGAGCAC<br>R: AGAGCTCCACTTCAGCGAAA	-3.534	0.972	91.9
ACT1	F: GGTGATGACGCTCCAAGA<br>R: TTGACCCATACCGACCATAA	-3.531	0.971	91.9

Table S3: Relative expression of amino acid permease genes in *B. bruxellensis* LAMAP2480 and LAMAP1359 strains under different amino acid conditions. All experiments were performed with three biological replicates, each measured in triplicate. Different letters within each gene column indicate significant differences among amino acid conditions for the same strain. Statistical differences were determined by one-way ANOVA followed by Fisher's LSD ($p < 0.05$).

Strain	Amino acid	*GNP1*	*GAP1*	*TAT1*
LAMAP2480	Pro	0.879 ± 0.003 (b)	0.582 ± 0.001 (b)	0.413 ± 0.014 (c)
	Trp	0.220 ± 0.006 (d)	0.202 ± 0.004 (d)	0.312 ± 0.020 (c)
	Gln	0.316 ± 0.010 (c)	0.668 ± 0.016 (a)	0.668 ± 0.015 (b)
	Arg	1.026 ± 0.012 (a)	0.441 ± 0.001 (c)	1.771 ± 0.095 (a)
LAMAP1359	Pro	2.616 ± 0.107 (a)	0.442 ± 0.040 (b)	0.689 ± 0.001 (c)
	Trp	2.192 ± 0.075 (b)	0.664 ± 0.043 (b)	3.852 ± 0.157 (a)
	Gln	1.496 ± 0.086 (c)	1.841 ± 0.132 (a)	2.034 ± 0.116 (b)
	Arg	0.412 ± 0.006 (d)	0.467 ± 0.009 (b)	1.840 ± 0.030 (b)
